# Transmission Properties of Human PrP 102L Prions Challenge the Relevance of Mouse Models of GSS

**DOI:** 10.1371/journal.ppat.1004953

**Published:** 2015-07-02

**Authors:** Emmanuel A. Asante, Andrew Grimshaw, Michelle Smidak, Tatiana Jakubcova, Andrew Tomlinson, Asif Jeelani, Shyma Hamdan, Caroline Powell, Susan Joiner, Jacqueline M. Linehan, Sebastian Brandner, Jonathan D. F. Wadsworth, John Collinge

**Affiliations:** MRC Prion Unit and Department of Neurodegenerative Disease, UCL Institute of Neurology, National Hospital for Neurology and Neurosurgery, London, United Kingdom; Creighton University, UNITED STATES

## Abstract

Inherited prion disease (IPD) is caused by autosomal-dominant pathogenic mutations in the human prion protein (PrP) gene (*PRNP*). A proline to leucine substitution at PrP residue 102 (P102L) is classically associated with Gerstmann-Sträussler-Scheinker (GSS) disease but shows marked clinical and neuropathological variability within kindreds that may be caused by variable propagation of distinct prion strains generated from either PrP 102L or wild type PrP. To-date the transmission properties of prions propagated in P102L patients remain ill-defined. Multiple mouse models of GSS have focused on mutating the corresponding residue of murine PrP (P101L), however murine PrP 101L, a novel PrP primary structure, may not have the repertoire of pathogenic prion conformations necessary to accurately model the human disease. Here we describe the transmission properties of prions generated in human PrP 102L expressing transgenic mice that were generated after primary challenge with *ex vivo* human GSS P102L or classical CJD prions. We show that distinct strains of prions were generated in these mice dependent upon source of the inoculum (either GSS P102L or CJD brain) and have designated these GSS-102L and CJD-102L prions, respectively. GSS-102L prions have transmission properties distinct from all prion strains seen in sporadic and acquired human prion disease. Significantly, GSS-102L prions appear incapable of transmitting disease to conventional mice expressing wild type mouse PrP, which contrasts strikingly with the reported transmission properties of prions generated in GSS P102L-challenged mice expressing mouse PrP 101L. We conclude that future transgenic modeling of IPDs should focus exclusively on expression of mutant human PrP, as other approaches may generate novel experimental prion strains that are unrelated to human disease.

## Author Summary

Inherited prion disease (IPD) is caused by pathogenic mutations in the human prion protein (PrP) gene leading to the formation of lethal prions in the brain. To-date the properties of prions causing IPD and their similarities to prions causing other forms of human prion disease remain ill-defined. In the present study we have investigated the properties of prions seen in patients with Gerstmann-Sträussler-Scheinker (GSS) disease associated with the substitution of leucine for proline at amino acid position 102 (GSS P102L). We examined the ability of these prions to infect transgenic mice expressing human mutant 102L PrP, human wild-type PrP or wild-type mice. We found that GSS-102L prions have properties distinct from other types of human prions by showing that they can only infect transgenic mice expressing human PrP carrying the same mutation. Mice expressing wild-type human PrP or wild-type mouse PrP were entirely resistant to infection with GSS-102L prions. We conclude that accurate modeling of inherited prion disease requires the expression of authentic mutant human PrP in transgenic models, as other approaches may generate results that do not mirror the human disease.

## Introduction

Prion diseases are a closely related group of neurodegenerative conditions which affect both humans and animals [1,2]. They are both experimentally and, in some cases, naturally transmissible within and between mammalian species. Cross-species transmission is generally much less efficient than within-species transmissions, being limited by a ‘species’ or transmission barrier [2,3]. Prion diseases in humans include Creutzfeldt-Jakob disease (CJD), Gerstmann-Sträussler-Scheinker disease (GSS), fatal familial insomnia (FFI), kuru and variant CJD (vCJD) [1,4,5].

According to the widely accepted ‘protein-only’ hypothesis [6], the central feature of prion disease is the conversion of host-encoded cellular prion protein (PrP^C^) to alternative isoforms designated PrP^Sc^ [1,2,7]. It is proposed that PrP^Sc^ is the infectious agent acting to replicate itself with high fidelity by recruiting endogenous PrP^C^ and that the difference between these isoforms lies purely in the monomer conformation and its state of aggregation [1,2,8] although it is now clear that infectivity can also be associated with protease-sensitive disease-related PrP assemblies distinct from classical PrP^Sc^ [9–11] and that infectious and neurotoxic PrP species can be uncoupled [12,13]. Inherited prion disease (IPD) is caused by autosomal-dominant mutations in the human PrP gene (*PRNP*) and constitute about 15% of all human prion disease [4,14]. Over 40 mutations have been identified, but the precise biochemical mechanisms that lead to disease remain unknown. Within the framework of the protein-only hypothesis, pathogenic mutations in PrP are thought to predispose the mutant proteins to adopt disease-causing conformations and assembly states [2–4].

A proline to leucine substitution at codon 102 (P102L) of human PrP is the most common mutation associated with the GSS phenotype and was first reported in 1989 [15]. Many other kindreds have now been documented worldwide [16], including the original Austrian family reported by Gerstmann, Sträussler, and Scheinker in 1936 [17,18]. Progressive ataxia is the dominant clinical feature, with dementia and pyramidal features occurring later in a disease course typically much longer than that of classical CJD. However, marked variability at both the clinical and neuropathological levels is apparent, with some patients developing a classical CJD-like phenotype with early and rapidly progressive dementia [18–30]. A significant part of this phenotypic variability appears to be contributed by variable propagation of distinct disease-related PrP species generated from either PrP 102L [21,22] or wild type PrP [24,27]. Two distinct abnormal conformers of PrP 102L that generate proteinase K (PK)-resistant fragments of either ~21–30 kDa or ~8 kDa [21,22,24,27,31] have distinct prion transmission properties in 101LL PrP gene knock-in mice [25], while the potential transmissibility or neurotoxicity of abnormal conformers of wild type PrP (that generate PK-resistant fragments of 21–30 kDa [24,27]) remains unknown. Such heterogeneity in disease-related PrP isoforms present in IPD P102L patient brain severely complicates interpretation of transmissions in both conventional and transgenic mice. The conformational selection hypothesis [2,32] predicts that heterogeneous prions formed from PrP in distinct conformations would be differentially selected by hosts expressing different PrP primary sequences. In this regard expression of the homotypic human mutant protein in the host may be critical to accurately model the disease, as only the human mutant protein may be conformationally susceptible to the prion strain involved [2,3,33]. Much of the transgenic modelling of inherited prion disease has however focused on superimposing human PrP mutations onto rodent PrP in order to establish whether infectious prions can be generated *de novo*. An extremely important consideration in such studies is whether superimposition of pathogenic human PrP mutation into mouse PrP will have the same structural consequences [2,3,33,34]. The possibility of propagating novel prion strains that do not recapitulate the molecular and neuropathological phenotype of the original human disease appears probable [2,3,33] and indeed has been documented with variant CJD transmissions [35].

Recently we established that IPD P102L patient brain isolates could transmit disease with 100% clinical attack rates and short incubation periods to transgenic mice expressing human PrP 102L on a mouse PrP null background (designated 102LL Tg27 mice) [33]. In these transmissions we observed the propagation of the abnormal conformer of PrP 102L that generates protease-resistant fragments of ~21–30 kDa [33]. We also demonstrated that such mice were susceptible to infection with classical CJD prions leading to the generation of prions with altered PrP^Sc^ glycoform ratios [33]. The availability of these prions from 102LL Tg27 mice, in which disease-related PrP is entirely composed of PrP 102L (as opposed to the heterogeneous PrP in primary human GSS brain inoculum), now permits direct testing of their host range and in particular the ability of these prions to propagate using wild type human PrP or mouse PrP as substrate. Our findings show that human PrP 102L can support the propagation of distinct prion strains and that human PrP 102L prions have transmission properties strikingly different from those generated in transmission models in which the 102L mutation was superimposed onto mouse PrP.

## Results

### Efficient transmission of GSS-102L prions on further passage in 102LL Tg27 mice

Prions originating from the primary transmission of three different IPD P102L patient brains to 102LL Tg27 mice [33] (hereafter designated GSS-102L prions) transmitted clinical prion disease with 100% attack rates and short mean incubation periods (~165 days) when passaged in further 102LL Tg27 mice (Table 1).

**Table 1:** Transmission of homogenous GSS-102L prions to wild type FVB mice or transgenic mice expressing PrP 102L or wild type human PrP.

Mouse Line	Inoculum code	Primary Inocula details*	Incubation period (days ± sem)	Clinical signs	IHC^†^	IB^†^	Total affected^‡^
**102LL Tg27**	I7110	IPD P102L I1087	166 ± 3	9/9	9/9	9/9	9/9^#^
	I7111	IPD P102L I1479	165 ± 3	9/9	9/9	9/9	9/9^#^
	I7112	IPD P102L I1480	164 ± 2	10/10	8/8	10/10	10/10^#^
**FVB/N**	I7110	IPD P102L I1087	>501	0/8	0/8	0/8	0/8^§^
	I7111	IPD P102L I1479	>603	0/5	0/5	0/5	0/5^§^
	I7112	IPD P102L I1480	>558	0/5	0/5	0/5	0/5^§^
**129MM Tg35c**	I7110	IPD P102L I1087	>526	0/7	0/7	0/7	0/7^§^
	I7111	IPD P102L I1479	>650	0/8	0/8	0/8	0/8^§^
	I7112	IPD P102L I1480	>593	0/8	0/8	0/8	0/8^§^
**129VV Tg152c**	I7110	IPD P102L I1087	>415	0/7	0/7	0/7	0/7^§^
	I7111	IPD P102L I1479	>368	0/10	0/10	0/10	0/10^§^
	I7112	IPD P102L I1480	>421	0/7	0/7	0/7	0/7^§^

IHC = immunohistochemistry; IB = immunoblotting. Tg35c and Tg152c are congenic on FVB/N genetic background.

* Three IPD P102L patient brain isolates were passaged in 102LL Tg27 transgenic mice to generate GSS-102L prions. Details of these primary transmission are described in reference [33].

^†^ All samples were analysed by both IB and IHC using monoclonal antibodies ICSM 18 and ICSM 35. In 102LL Tg27 tissues ICSM 35 serves as a control to define non-specific background.

^‡^ Positive either by clinical signs, immunoblotting and/or immunohistochemistry.

^#^ All samples were positive for ICSM 18 but negative for ICSM 35.

^§^ All samples were negative for both ICSM 18 and ICSM 35.

**Fig 1:**
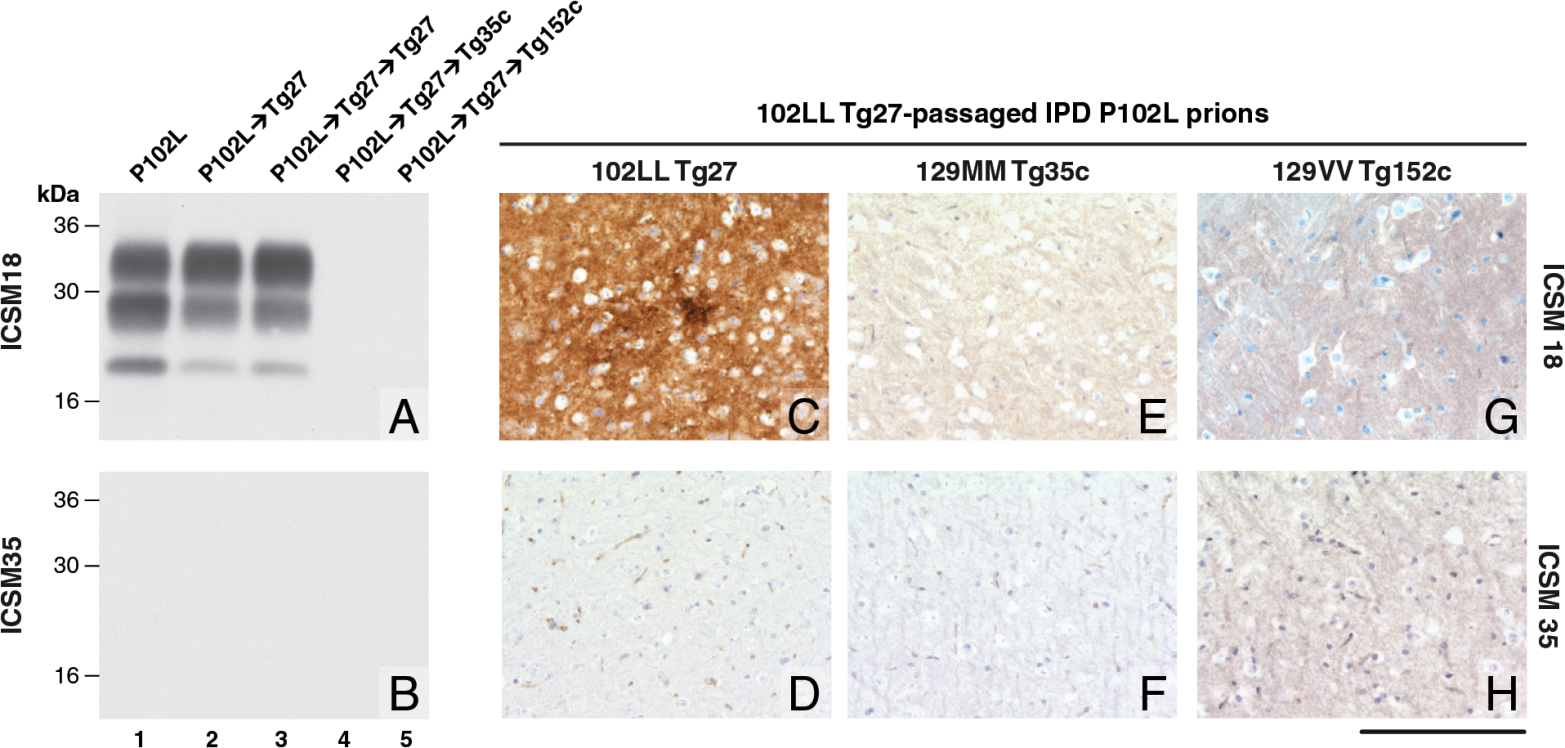
Detection of disease-related PrP by immunohistochemistry and immunoblotting in brains of transgenic mice inoculated with GSS-102L prions. The anti-PrP monoclonal antibodies used were, ICSM 18 (panels A, C, E, G) that recognizes both human PrP 102L and wild type human PrP and ICSM 35 (panels B, D, F, H) that recognizes wild type human PrP but not human PrP102L. The provenance of each brain sample analysed by immunoblotting is designated above each lane in panels A and B. (A, B) IPD P102L patient brain homogenate (P102L) in comparison to primary and secondary transmission into transgenic mice. All transgenic lines designated above each panel (C, E, G) were inoculated with inherited prion disease *PRNP* P102L 129 MM (IPD P102L) prions previously passaged in 102LL Tg27 transgenic mice. Only 102LL Tg27 transgenic mice were susceptible to the homogeneous 102L prions. Scale bar, 160 μm in all images.

Brain samples of all mice in these transmissions were positive for PK-resistant PrP 102L by immunoblotting using ICSM 18 (Fig 1A) (Table 1).

Both the PK-resistant PrP fragment size (~21–30 kDa) and predominance of the di-glycosylated PrP glycoform mirrored that seen in the 102LL Tg27 mouse brain inoculum (Fig 1A). Immunohistochemistry with ICSM 18 showed extensive abnormal PrP deposition throughout the brain (thalamus shown in Fig 1C) accompanied by prominent astrocytosis and spongiosis. Collectively, these findings establish that IPD P102L prions propagate with high efficiency when serially passaged in 102LL Tg27 mice.

### GSS-102L prions fail to transmit prion infection to wild type mice or transgenic mice expressing wild type human PrP

Much of the previous modeling of IPD P102L has involved superimposing the mutation onto the wild type mouse PrP sequence (reviewed in ref [3]). However, it is unclear if challenge of mouse PrP 101L with human P102L prions would lead to the generation of authentic human prion strains or conversely would lead to the generation of experimental prion strains with different transmission characteristics. Notably, after human P102L prions were passaged once in 101LL PrP gene knock-in mice the resultant prions were shown to readily infect wild type mice [36]. We therefore inoculated the GSS-102L prion isolates that transmitted efficiently on passage in 102LL Tg27 mice to wild type mice and also to transgenic mice expressing wild type human PrP on a mouse PrP null background. Strikingly, in complete contrast to findings with the mouse 101LL PrP gene knock-in model we found that GSS-102L prions failed to produce clinical prion disease or any evidence of sub-clinical prion infection when inoculated into wild type mice (Table 1). Even more remarkably the same GSS-102L prions produced no clinical prion disease or evidence of sub-clinical prion infection when inoculated into transgenic mice expressing wild type human PrP (Table 1). In all of these negative transmissions examination of brain showed no detectable PrP^Sc^ by high sensitivity immunoblotting (Fig 1A and 1B, lanes 4 and 5) or abnormal PrP deposition by immunohistochemistry (Fig 1E–1H).

In addition, no evidence for elevated levels of spongiosis or gliosis in comparison to the brain of uninoculated age-matched control mice was observed. Collectively these data establish that GSS-102L prions which replicate with high efficiency in a host expressing human PrP 102L are unable to propagate using wild type human PrP or wild type mouse PrP as substrate.

### CJD-102L prions are distinct from GSS 102L prions

In comparison to IPD P102L prions, transmission of classical CJD prions to 102LL Tg27 mice appears to be limited by a transmission barrier [33]. In primary transmissions, although nearly all CJD prion-challenged 102LL Tg27 mice showed evidence for prion infection, only a proportion of mice developed clinical prion disease and then only after prolonged incubation periods [33]. In addition, a change in propagated PrP^Sc^ type was observed (which itself is indicative of a transmission barrier [2]) with PrP^Sc^ glycoform ratios switching from those present in the CJD inocula to ones that more closely resemble those seen in the brain of IPD P102L patients and IPD P102L prion-challenged Tg27 mice [33]. From these primary transmissions, we were unable to distinguish whether the 102L mutation in the host PrP had directly dictated the strain characteristics of the propagated prions (to essentially become congruent with GSS-102L prions) or whether CJD-like prion strain properties were retained. To investigate this, we passaged prions from CJD-challenged 102LL Tg27 mice (hereafter designated CJD-102L prions) in further 102LL Tg27 mice, in transgenic mice expressing wild type human PrP and in wild type mice (Table 2).

**Table 2:** Serial transmission of CJD-102L prions in transgenic mice homozygous for 102L or wild type human PrP.

Mouse Line	Inoculum code	Primary inocula details*	Incubation period (days ± sem)	Clinical signs	IHC^†^	IB^†^	Total affected^#^
**102LL Tg27**	I7113	sCJD T1MM →102LL Tg27	574	0/9	3/9	9/9	9/9^‡^
	I7114	iCJD T2 MM (DM) →102LL Tg27	631 ± 12	7/7	6/7	7/7	7/7^‡^
	I7115	iCJDT3 MV (GH) →102LL Tg27	387 ± 11	6/8	8/8	8/8	8/8^‡^
	I7116	iCJDT3 VV (GH) →102LL Tg27	373 ± 6	4/8	8/8	8/8	8/8^‡^
**FVB/N**	I7113	sCJD T1MM →102LL Tg27	>488	0/6	0/6	0/6	0/6
	I7114	iCJD T2 MM (GH)→102LL Tg27	761	1/5^§^	ND	1/1	1/1
	I7115	iCJDT3 MV (GH) →102LL Tg27	>536	0/5	0/5	0/5	0/5
	I7116	iCJDT3 VV (GH) →102LL Tg27	>536	0/6	0/6	0/6	0/6
**129MM Tg35c**	I7113	sCJD T1 MM →102LL Tg27	322 ± 10	9/10	9/9	10/10	10/10
	I7114	iCJD T2 MM (DM)→102LL Tg27	255 ± 4	8/8	8/8	8/8	8/8
	I7115	iCJDT3 MV (GH) →102LL Tg27	627 ± 14	8/8	8/8	8/8	8/8
	I7116	iCJDT3 VV (GH) →102LL Tg27	696 ± 11	6/7	6/7	6/7	6/7
**129VV Tg152c**	I7113	sCJD T1 MM →102LL Tg27	330 ± 36	6/8	7/8	7/7	8/8
	I7114	iCJD T2 MM (DM)→102LL Tg27	179 ± 11	8/8	8/8	8/8	8/8
	I7115	iCJDT3 MV (GH) →102LL Tg27	148, 196	2/2	2/2	2/2	2/2
	I7116	iCJDT3 VV (GH) →102LL Tg27	216 ± 16	7/7	5/7	7/7	7/7

IHC = immunohistochemistry; IB = immunoblotting; ND = not determined; Tg35c and Tg152c are congenic on FVB/N genetic background; sCJD = sporadic CJD; iCJD = iatrogenic CJD; DM, = dura mater; GH = growth hormone.

* All inocula were first passaged in 102LL Tg27 transgenic mice to generate CJD-102L prions.

^†^ All samples were analysed by both IB and IHC using monoclonal antibodies ICSM 18 and ICSM 35. In 102LL Tg27 tissues ICSM 35 serves as a control to define non-specific background.

^#^ Positive either by clinical signs, immunoblotting and/or immunohistochemistry.

^‡^ All samples were positive for ICSM 18 but negative for ICSM 35.

^§^ Only 1 sample was available for analysis by IB.

**Fig 2:**
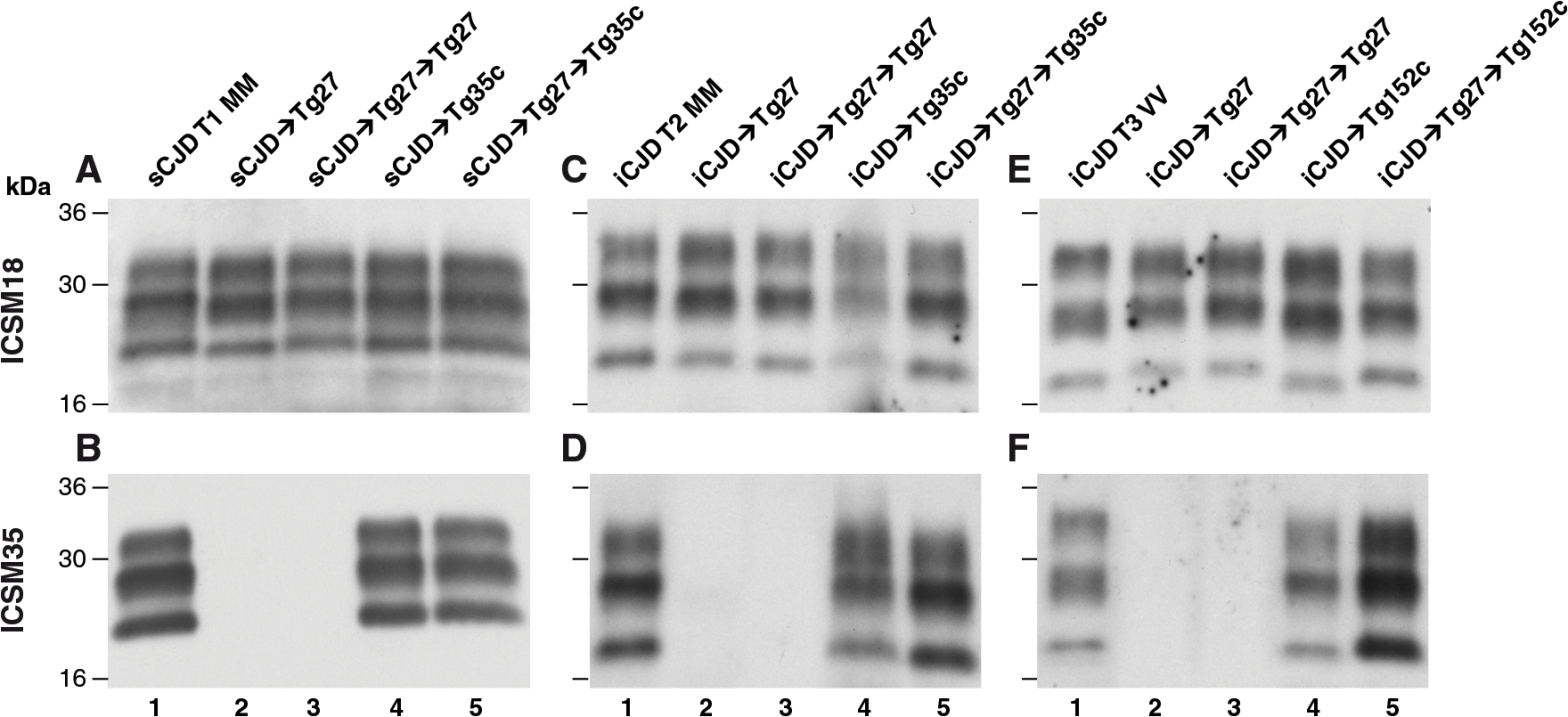
Immunoblot detection of PrP^Sc^ in proteinase K-digested human and transgenic mouse brain homogenates using enhanced chemiluminescence. The anti-PrP monoclonal antibodies used were, ICSM 18 (panels A, C, E) that recognizes both PrP 102L and wild type PrP and ICSM 35 (panels B, D, F) that recognizes wild type PrP but not PrP102L. The provenance of each brain sample is designated above each lane. (A, B) Sporadic CJD *PRNP* 129MM with type 1 PrP^**Sc**^ patient brain homogenate (sCJD T1 MM). (C, D) Iatrogenic CJD *PRNP* 129MM with type 2 PrP^**Sc**^ patient brain homogenate (iCJD (DM) T2 MM) in comparison to primary and secondary transmission into transgenic mice. (E, F) Iatrogenic CJD *PRNP* 129VV with type 3 PrP^**Sc**^ patient brain homogenate (iCJD (GH) T3 VV) in comparison to primary and secondary transmission into transgenic mice. Transgenic mice are 102LL Tg27 (Tg27), 129MM Tg35c (Tg35c) and 129VV Tg152c (Tg152c). DM, dura mater; GH, growth hormone.

**Fig 3:**
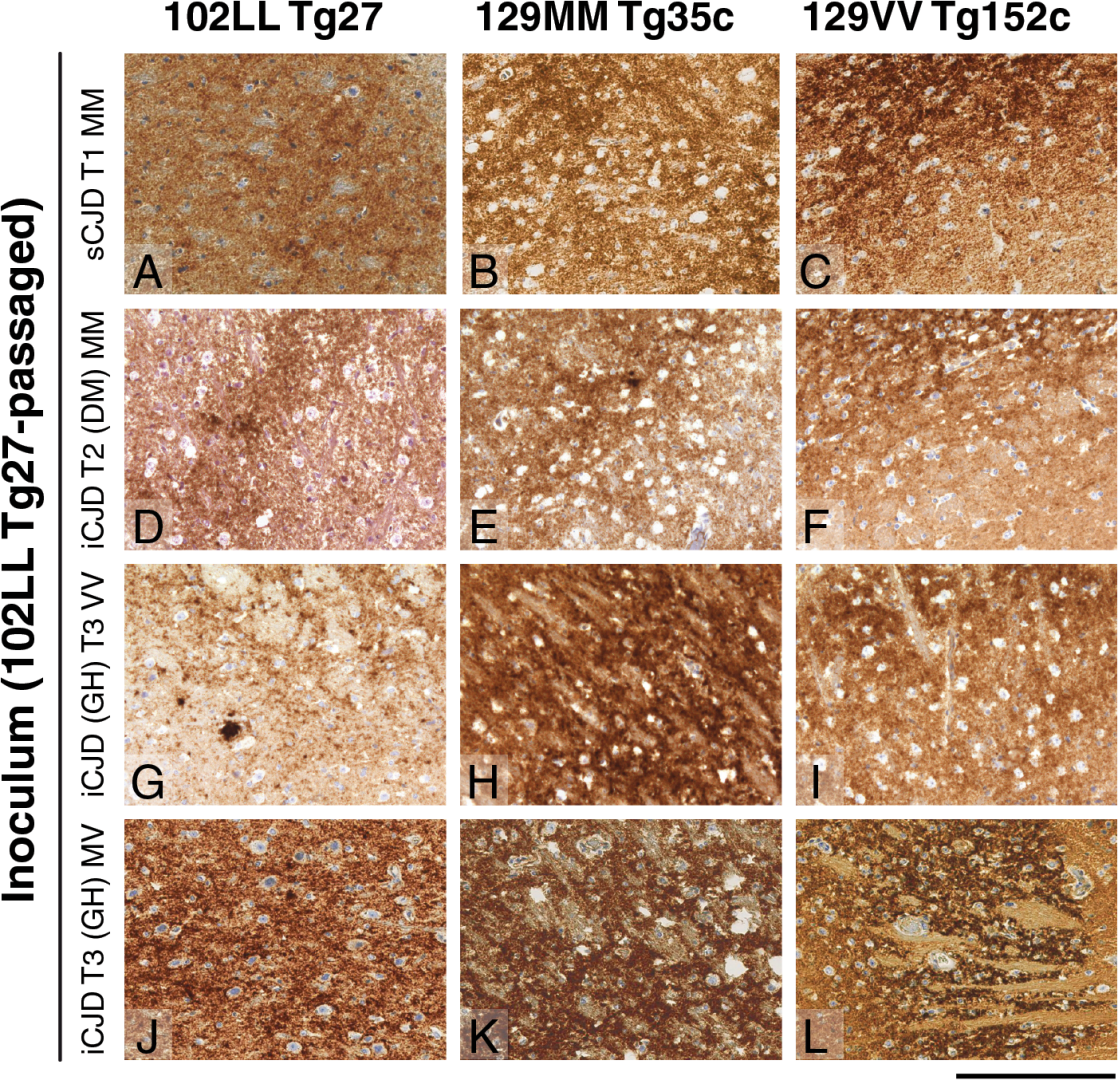
Immunohistochemical detection of abnormal PrP deposition in brains of transgenic mice challenged with CJD-102L prions. All images show abnormal PrP deposition in the thalamus stained with anti-PrP monoclonal antibody ICSM 18. The inoculum and line of transgenic mouse is designated to the left and above the panels respectively. All inocula were from primary transmissions of classical CJD prions from patient brain to 102LL Tg27 mice; sporadic CJD *PRNP* 129MM with type 1 PrP^**Sc**^ (sCJD T1 MM) (A-C), iatrogenic CJD *PRNP* 129MM with type 2 PrP^**Sc**^ (iCJD T2 (DM) MM) (D-F), iatrogenic CJD *PRNP* 129VV with type 3 PrP^**Sc**^ (iCJD (GH) T3 VV) (G-I), iatrogenic CJD *PRNP* 129MV with type 3 PrP^**Sc**^ (iCJD T3 (GH) MV) (J-L) DM, dura mater; GH, growth hormone. Scale bar, 160 μm in all images.

In 102LL Tg27 mice we observed that the barrier to development of clinical prion disease seen at primary transmission of classical CJD prions was not abrogated at secondary passage (Table 2). Although nearly all CJD-102L prion-inoculated mice developed prion infection, as evidenced by detection of PrP^Sc^ (Fig 2) and abnormal PrP deposition throughout the brain (Fig 3), clinical prion disease was again only observed in a proportion of inoculated recipients and then only at prolonged incubation periods (Table 2). PrP^Sc^ typing showed that the altered PrP^Sc^ glycoform ratio of CJD-102L prions generated after primary transmission of classical CJD prions to 102LL Tg27 mice was not maintained after further passage in the same mice. The PrP^Sc^ type now appeared to more closely resemble the original classical CJD inoculum with a predominance of mono-glycosylated PrP and was readily distinguishable from the di-glycoslyated PrP dominant glycoform pattern seen after secondary passage of GSS 102L prions in 102LL Tg27 mice (Fig 2A, 2C and 2E) (Table 3). From these transmissions we concluded that GSS-102L prions and CJD-102L prions have incongruent transmission properties after further passage in 102LL Tg27 mice.

**Table 3:** Glycoform analysis of Tg27-passaged classical CJD (CJD-102L) and IPD P102L (GSS-102L) prions transmitted to transgenic 102LL Tg27 and 129MM Tg35c mice.

	Classical CJD T2 MM	T2 MM/ Tg27*	T2 MM/Tg27/Tg27	T2 MM/ Tg27/Tg35c^†^	P102L/Tg27/Tg27
Diglycosylated PrPSc	19.6 ± 1^‡^	50.8 ± 5.0	34.3 ± 0.4	32.2 ± 2.0	61.5 ± 1.4
		P< 0.0001	P< 0.0001	P< 0.0001	P< 0.0001
Monoglycosylated PrP^Sc^	47.9 ± 1	36.5 ± 2.0	42.5 ± 1.9	41.5 ± 1.1	29.4 ± 0.8
		P< 0.0001	P< 0.01	P< 0.002	P< 0.0001
Unglycosylated PrP^Sc^	32.6 ± 1	12.6 ± 3.0	23.2 ± 2.0	26.2 ± 1.0	26.2 ± 1.0
		P< 0.0001	P< 0.001	P< 0.007	P< 0.0001

* Tg27 = 102LL Tg27

^†^ Tg35c = 129MM Tg35 mice congenic on FVB/N genetic background.

^‡^ Glycoform ratios of PrP^Sc^ propagated in transgenic mice (n = 4 per line) inoculated with Tg27-passaged classical CJD and IPD P102L prions has been compared with the human T2 MM glycoform ratio (n = 11) [31]. Data show mean ± sem. *P* values relate to comparison with classical CJD T2 MM (unpaired two-tailed *t*-test).

Importantly, the disparate nature of CJD-102L prions and GSS-102L prions became obvious after examining the transmission properties of CJD-102L prions in transgenic mice expressing wild type human PrP. In complete contrast to GSS-102L prions, all CJD-102L prion isolates transmitted clinical prion disease to mice expressing wild type human PrP in a fashion analogous to the original CJD inoculum (Table 2). In these transmissions PrP^Sc^ was readily detected in brain by immunoblotting (Fig 2) and abnormal PrP deposition was observed throughout the brain by immunohistochemistry (Fig 3).

Humanised transgenic mice expressing human PrP 129 valine on a *Prnp* null background are highly susceptible to sporadic CJD prions regardless of the PrP^Sc^ type or codon 129 genotype of the inoculum [37–43]. These transmissions are typically characterised by 100% attack rates of prion infection producing uniform clinical prion disease after similar short incubation periods of around 200 days. The absence of a transmission barrier to sporadic CJD prions is not however uniformly observed in transgenic mice expressing human PrP 129 methionine on a *Prnp* null background. Here mismatch at residue 129 between the inoculum and host can significantly affect transmission [41,44–46] as evidenced by more prolonged and variable incubation periods and reduced attack rates [41,43,44]. Remarkably, we observed that CJD-102L prions behaved in a closely similar fashion that corresponded with the codon 129 status of the original CJD inoculum (Table 2). This was striking because all of the CJD-102L prion isolates have PrP with residue 129 methionine. Consistent with the CJD-like transmission properties of CJD-102L prions in transgenic mice expressing wild type human PrP, PrP^Sc^ typing of the recipient mouse brain showed that the di-glycosylated dominant PrP^Sc^ glycoform ratio of CJD-102L prions in the inoculum had switched to a mono-glycosylated PrP^Sc^ dominant pattern which more closely resemble CJD prions (Table 3; Fig 2B, 2D and 2F, lanes 5). Collectively, these data show that CJD-102L prions are distinct from GSS-102L prions and retain the transmission properties of the original CJD prion strains. Notwithstanding these observations, all the CJD-102L prion isolates were obtained after a single passage of classical CJD prions in 102LL Tg27 mice and it remains to be seen whether serial passage on the mutated sequence would lead to similar conservation of CJD phenotype.

Consistent with the finding that classical CJD prions transmit prion infection only occasionally to wild type mice with long and variable incubation periods [37,39,40,42,47] we found that CJD-102L prions were also unable to propagate efficiently in wild type mice (Table 2). We found that only one out of eighteen CJD-102L prion-inoculated wild type mice became infected (Table 2) with all other mice showing no evidence of subclinical prion infection by either PrP immunoblotting or immunohistochemistry.

## Discussion

Co-propagation of distinct disease-related PrP conformers in IPD brain, combined with differences in their neuropathological targeting, abundance and potential neurotoxicity, provides a general molecular mechanism underlying phenotypic heterogeneity in patients with the same *PRNP* mutation. Previously we and others have reported the propagation of distinct isoforms of protease-resistant PrP with divergent properties in IPD P102L patient brain and such molecular heterogeneity severely hampers interpretation of primary transmissions to both conventional and transgenic mice (for review see [3]).

**Fig 4:**
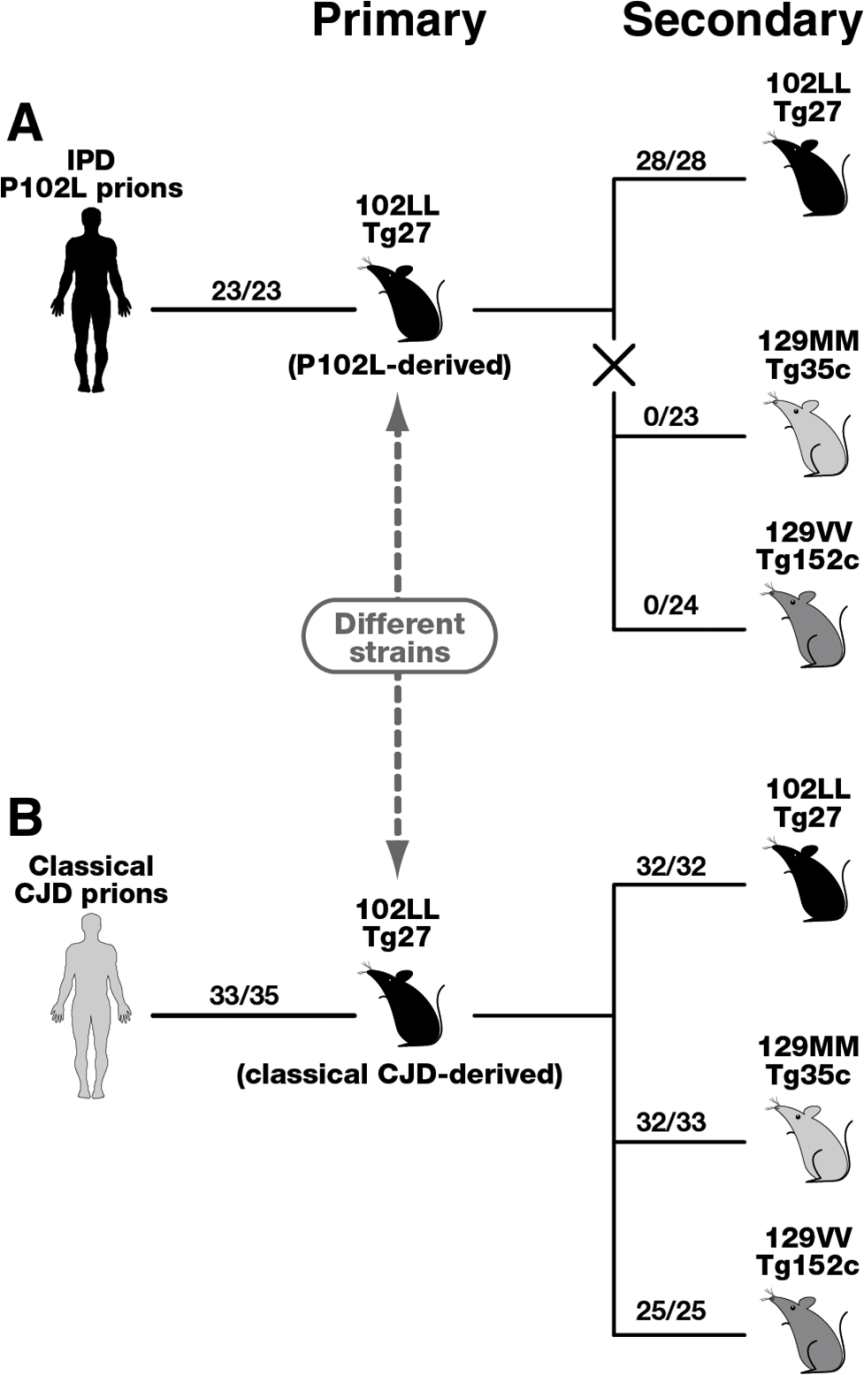
Summary of serial passages of IPD P102L prions (A) and classical CJD prions (B) to transgenic mice expressing human PrP 102L or wild type human PrP. The total number of prion-affected mice (both clinically and sub-clinically infected) is reported for each inoculated group: 102LL Tg27 mice (black), 129MM Tg35c mice (light grey) and 129VV Tg152c mice (dark grey). Animals were scored by clinical signs, immunoblotting for PrP^**Sc**^, and/or PrP immunohistochemistry. The X in panel A denotes the marked transmission barrier that prevents GSS-102L prions from propagating in transgenic mice expressing wild-type human PrP. Primary transmissions of P102L and classical CJD prions have been published previously [33].

In the present study we have investigated the properties of prions generated in transgenic mice expressing human PrP 102L following the intracerebral inoculation of IPD P102L or classical CJD brain isolates. The resultant prion isolates from these transgenic mouse brain were designated GSS-102L or CJD-102L prions, respectively, and because they are associated exclusively with disease-related conformers of human PrP 102L this enables unequivocal examination of the effects that this point mutation has on prion transmission barriers. Our findings show that GSS-102L and CJD-102L prions are distinct from one another with divergent prion strain transmission properties following further passage in transgenic mice expressing either human PrP 102L or wild type human PrP (Fig 4). Thus human PrP 102L is capable of supporting the propagation of distinct lethal prion strains and these data establish that the point mutation does not restrict PrP 102L to a single dominant pathogenic assembly state when templated by an exogenous prion strain.

Importantly our model has enabled us to isolate and investigate the transmission properties of prions originating from IPD P102L patient brain following amplification exclusively on human 102L PrP. Our data show that 102L PrP prions from IPD P102L patient brain that generate PK-resistant PrP fragments of ~21–30 kDa have prion strain transmission properties distinct from all other prion strains propagated in acquired or sporadic human prion disease. The most outstanding feature of this prion strain is its inability to propagate in transgenic mice expressing wild type PrP. Significantly, the inability of GSS-102L prions to also propagate in wild type mice clearly shows that this prion strain is distinct from prions generated in IPD P102L prion-challenged 101LL PrP gene knock-in mice [36]. The remarkable ease of transmission of 101L-passaged IPD P102L prions to wild type mice [36] contrasts strikingly with our data and suggests that a novel prion strain was propagated by the mutant mouse PrP rather than faithful replication of the authentic human PrP 102L prion strain. We therefore recommend that future transgenic modeling of inherited prion disease should focus exclusively on using models that express the homotypic mutant human PrP primary sequence.

We and others have reported that variable involvement of disease-related conformers of wild type human PrP may contribute to phenotypic heterogeneity in IPD P102L [24,27]. However the mechanism by which abnormal wild type PrP is deposited in P102L patient brain remains ill-defined. Wild type PrP may be recruited by a seeded reaction with 102L PrP^Sc^ or may accumulate independently as a consequence of pathological changes associated with disease progression. Notably, the glycoform ratios of proteinase K-resistant fragments of 102L PrP and wild type PrP from P102L patient brain are distinct from each other [24] [27] suggesting that the 102L point mutation powerfully dictates thermodynamic preferences for disease-related PrP assembly states that cannot be adopted by wild type PrP and that a significant transmission barrier may be associated with conversion of wild type PrP by a 102L PrP^Sc^ seed. This idea is supported by the observation that PK-resistant wild-type PrP in P102L patient brain does not appear to exceed approximately 10% of total PK-resistant PrP [24,27]. Here we show that GSS-102L prions that propagate efficiently in further 102LL Tg27 transgenic mice fail to produce prion infection in transgenic mice expressing wild type human PrP. Based upon the strength of this transmission barrier we conclude that seeded conversion of wild type PrP by abnormal conformers of 102L PrP that generate proteolytic fragments of ~ 21–30 kDa may, at best, be highly inefficient. From these data it is tempting to speculate that abnormal conformers of 102L PrP that generate protease-resistant fragments of 8 kDa might instead be responsible for variable recruitment of wild type PrP in IPD P102L patient brain. However other explanations may be equally possible. In particular, our transmission experiments do not mirror the situation in IPD P102L patient brain where both PrP 102L and wild type PrP are co-expressed. Thus in IPD P102L patient brain, wild type PrP will be exposed throughout the disease time course to all propagating 102L PrP^Sc^ species (rather than only at inoculation) and such prolonged exposure *in vivo* may be required for the generation of misfolded isoforms of wild type PrP. Alternatively because prion strains appear to comprise a quasispecies maintained under host selection pressure (rather than constituting a single molecular clone) [2,48–50] minor subtypes of 102L PrP^Sc^ may be populated differently in individual P102L patients leading to variable degrees of recruitment of wild type PrP. Notwithstanding such possibilities, at present we cannot conclusively resolve whether wild type PrP in IPD P102L patient brain misfolds through a directly seeded conversion reaction with an abnormal 102L PrP template or as a consequence of other pathological changes in the brain. In this regard, transmission experiments in heterozygous transgenic mice expressing both 102L PrP and wild type PrP would not be able to differentiate between these possibilities. Although the mechanism that leads to the accumulation of abnormal wild type PrP continues to remain ill-defined, this remains a potentially important contributor to phenotypic variation, not only in IPD P102L, but also in IPD associated with other *PRNP* mutations [51–56].

## Methods

### Ethics statement

Storage and biochemical analyses of post-mortem human brain samples and transmission studies to mice were performed with written informed consent from patients with capacity to give consent. Where patients were unable to give informed consent, assent was obtained from their relatives in accordance with UK legislation and Codes of Practice. Samples were stored and used in accordance with the Human Tissue Authority Codes of Practice and in line with the requirements of the Human Tissue Authority licence held by UCL Institute of Neurology. This study was performed with approval from the National Hospital for Neurology and Neurosurgery and the UCL Institute of Neurology Joint Research Ethics Committee (now National Research Ethics Service Committee, London—Queen Square)—REC references: 03/N036, 03/N038 and 03/N133. Work with mice was performed under approval and licence granted by the UK Home Office (Animals (Scientific Procedures) Act 1986; Project Licence number 70/6454) and conformed to University College London institutional and ARRIVE guidelines (www.nc3rs.org.uk/ARRIVE/).

### Transgenic mice

Transgenic mice homozygous for a human PrP^102L,129M^ transgene array and murine PrP null alleles (*Prnp* ^*o/o*^) designated Tg(HuPrP^102L 129M+/+^ *Prnp* ^*o/o*^)-27 mice (102LL Tg27) [33] have been described previously and were used without modification. Transgenic mice homozygous for a wild type human PrP^129M^ transgene array and murine PrP null alleles (*Prnp* ^*o/o*^) designated Tg(HuPrP^129M+/+^ *Prnp* ^*o/o*^)-35 congenic (129MM Tg35c) were derived by subjecting previously described 129MM Tg35 mice [35,44,57] to commercially available speed congenic backcrossing on FVB/N genetic background (Charles River UK). Similarly, transgenic mice homozygous for a wild type human PrP^129V^ transgene array and murine PrP null alleles (*Prnp* ^*o/o*^) designated Tg(HuPrP^129V+/+^ *Prnp* ^*o/o*^)-152 congenic (129VV Tg152c) were derived by subjecting previously described 129VV Tg152 mice [37,39,42] to the speed congenic scheme (Charles River UK). Inbred FVB/NHsd mice were supplied by Harlan UK Ltd.

### Transmission studies

Strict bio-safety protocols were followed. Inocula were prepared, using disposable equipment for each inoculum, in a microbiological containment level 3 laboratory and inoculations performed within a class 1 microbiological safety cabinet. Ten mice per group from three transgenic lines, 102LL Tg27, 129MM Tg35c, 129VV Tg152c and FVB/N wild type mice were inoculated with a panel of prion isolates, all previously passaged in 102LL Tg27 transgenic mice and therefore adapted to human 102L PrP. The primary inocula comprised human brain homogenates from three IPD P102L patients, one sporadic CJD patient and three iatrogenic CJD patients. Diagnosis of all cases had been neuropathologically confirmed. The genotype of each mouse was confirmed by PCR of DNA prior to inclusion and all mice were uniquely identified by sub-cutaneous transponders. Disposable cages were used and all cage lids and water bottles were also uniquely identified by transponder and remained with each cage of mice throughout the incubation period. Care of the mice was according to institutional and ARRIVE guidelines. Mice were anaesthetised with a mixture of halothane and O_2_, and intra-cerebrally inoculated into the right parietal lobe with 30 μl of 1% (w/v) brain homogenate prepared in Dulbecco’s phosphate buffered saline lacking Ca^2+^ or Mg^2+^ ions (D-PBS). All mice were thereafter examined daily for clinical signs of prion disease. Mice were killed if they exhibited any signs of distress or once a diagnosis of prion disease was established. At post-mortem brains from inoculated mice were removed, divided sagittally with half frozen and half fixed in 10% buffered formol saline.

### Antibodies

Anti-PrP monoclonal antibodies ICSM 18 and ICSM 35 were supplied by D-Gen Ltd, London, UK. ICSM antibodies were raised in *Prnp* ^*o/o*^ mice against α or β PrP as described elsewhere [58]. ICSM 18 is an IgG_1_ with an epitope spanning residues 142–153 of human PrP [58]. ICSM 35 is an IgG_2b_ with an epitope spanning residues 93–105 of human PrP [58,59]. ICSM 18 recognizes both human PrP 102L and wild type human PrP whereas ICSM 35 recognizes wild type human PrP but not human PrP 102L [24].

### Immunoblotting

Brain homogenates (10% (w/v)) were prepared in D-PBS and aliquots analysed in duplicate with or without proteinase K digestion (50 μg/ml final protease concentration, 1h, 37°C) by electrophoresis and immunoblotting as described previously [60,61]. Duplicate blots were blocked in PBS containing 0.05% v/v Tween-20 (PBST) and 5% w/v non-fat milk powder and probed with ICSM 18 or ICSM 35 anti-PrP monoclonal antibodies (0.2 μ g/ml final concentration in PBST) in conjunction with anti-mouse IgG-alkaline phosphatase conjugated secondary antibody and chemiluminescent substrate CDP-Star (Tropix Inc, Bedford, MA, USA) and visualized on Biomax MR film (Kodak) as described [60,61]. For analysis of PrP glycoforms, blots were developed in chemifluorescent substrate (AttoPhos; Promega) and visualized on a Storm 840 phosphoimager (Amersham) using ImageQuaNT software (Amersham) [31,61].

### Immunohistochemistry

Fixed brain was immersed in 98% formic acid for 1 h and paraffin wax embedded. Serial sections of 4 μm nominal thickness were pre-treated with Tris-Citrate EDTA buffer for antigen retrieval [61]. PrP deposition was visualized using ICSM 35 or ICSM 18 as the primary antibody, using an automated immunostaining system (www.ventana.com). Visualization was accomplished with diaminobenzidine staining. Bright field photographs were taken on an ImageView digital camera (www.soft-imaging.de) and composed with Adobe Photoshop.
